# Nutrition and Tuberculosis
*Reprinted (with additions) by permission from the *Transactions* of the Twenty-third Annual Conference of the National Association for the Prevention of Tuberculosis, Bristol, 2nd July, 1937.


**Published:** 1937

**Authors:** J. A. Nixon

**Affiliations:** Emeritus Professor of Medicine, University of Bristol


### The Bristol Medico-Chirurgical Journal

"*Scire est nescire, nisi id me*
*Scire alius sciret.*"

AUTUMN, 1937.

### NUTRITION AND TUBERCULOSIS.*

BY

Professor J. A. Nixon, C.M.G., M.D., F.R.C.P.,
*Emeritus Professor of Medicine, University of Bristol.*

It seems appropriate that "Nutrition and Tuberculosis" should be the subject of discussion in Bristol because the water of the Clifton Hotwells had a reputation for curing patients of consumption. Thus there was an early interest in this neighbourhood in tuberculosis, and as for nutrition we can read the advice that John Locke, the great philosopher, who was born at Wrington, gave to a foreigner visiting England. He said: "At Bristol see the Hot Well, St. George's Cave, where the Bristol diamonds are found, Redcliffe Church, and at Kingswood the

* Reprinted (with additions) by permission from the *Transactions* of the Twenty-third Annual Conference of the National Association for the Prevention of Tuberculosis, Bristol, 2nd July, 1937.

coal pits." Then he went on to more important topics: "Taste there the Milford oysters, marrow puddings . . . Bristol elvers, sherry, sack (which, with sugar, is called Bristol milk) and some other wines which, perhaps, you will not taste so good in London."

Nutrition is the sum total of the processes by which the nourishment of the body is effected. These processes include the constitution of the food, both qualitative and quantitative, the adequate oxygenization of the blood and the tissues, the efficiency of the processes of digestion, absorption, assimilation, circulation and excretion, proper exercises of the body, rest and sleep, freedom from worry, anxiety and emotional disturbance, general hygiene, freedom from disease, sunlight, fresh air, and inherited characters. Chief among these is food of a constitution that provides all the elements and complexes of sufficient quantity and quality needed for normal nutrition. There may be, as Holt said, normal nutrition without normal health, but there cannot be normal health without normal nutrition.

It will be realized that the open-air school can furnish several of the nutritional factors I have mentioned. In particular it provides fresh air, sunlight, hygiene, rest, proper exercise, and some degree of freedom from worry and emotional disturbance. Moreover, as open-air schools are generally conducted arrangements are made for giving meals to the scholars which are adequate both in quantity and quality. Every child attending the open-air schools in Bristol receives breakfast, dinner and tea at the schools.

In the Report of the Chief Medical Officer of the

Board of Education (1932) the results of open-air education are summed up thus:

"To the School Medical Officer open-air education is a direct means of preventive medicine. The anæmic child improves in regard to the hæmoglobin content of the blood, the emaciated child increases in weight, enlarged glands diminish or disappear, lung troubles improve and even vanish. The individual attention given to the child by the nurse and teacher, the opportunities for bathing and personal hygiene, the adequate meals, the rest hour and the special arrangements for physical training engender and foster habits of personal cleanliness and health difficult to obtain in the crowded conditions of the ordinary day school, and in this way open-air education tends to restore the enfeebled body to a normal condition of nutrition and energy, helps to dispel many of the nervous conditions incidental to child life in towns, and serves as a most valuable adjunct in the prevention of all forms of constitutional disease including tuberculosis."

As one reads carefully through this summary one is inclined to pause and ask: How much do these advantages really depend upon the education being in the "open-air"? It seems unreasonable to confine these advantages to such children as are found by medical examination to be "delicate." At present I am told that in Bristol, at any rate, only "delicate" children can attend an open-air school. I have asked if any Act of Parliament defines what is a "delicate" child. I have looked in the Oxford Dictionary for a medical interpretation of "delicate." The nearest I can find is "easily injured," "liable to illness."

As a parent as well as a doctor I am inclined to say that every child comes under this definition, "easily injured—liable to sickness."

So far as the school-child is concerned the open-air education appears to be so designed as to secure the maximum of those nutritional factors which I have already enumerated.

Unfortunately for some children, holidays intervene with detrimental effects. In many instances these children have been selected to attend open-air schools on account of their poor state of nutrition. In the holidays they may go back to homes where the feeding, the rest and the hygiene cannot be anything like the same as they were accustomed to getting in term-time at school. The standard of nutrition of many of these children then declines and has to be built up again in each term when they come back to school.

But those factors which are mainly environmental formed the basis of yesterday's discussion.

To-day I want to say a few words on the food-factor in nutrition with special reference to the prevention and treatment of tuberculosis.

It would be a grave mistake to allow the idea to prevail that dietetic measures by themselves will prevent or cure tuberculosis.

In Great Britain there is some tendency to discount the part played by malnutrition in the production of tuberculosis. Bently, investigating the problem in young unmarried women of the clerk, assistant, domestic and dress-making classes in London, concluded that diet played little or no part in the incidence of the disease. Yet the incidence of pulmonary tuberculosis is high amongst young stable-lads. Schultze and Zilva found that there was insufficient evidence to prove that diets deficient in fat-soluble vitamins rendered laboratory animals more susceptible to tuberculous inoculation; they found also that feeding inoculated guinea-pigs with cod-liver oil did not prolong their lives, improve their weight-curves, or curtail the extent of the disease.

Nevertheless, it would be unwise to conclude that under-feeding plays no part in determining failure of resistance to an infection which in this country is so prevalent that it is probable that every individual over the age of fourteen years has been infected with tuberculosis. Whether the infection is sufficient to produce symptoms of disease is dependent on the individual's resistance.

Guild and Green are convinced that the low economic status of the negro in the United States renders him disproportionately prone to tuberculosis. "Tuberculosis," writes Guild, "in the negro, as in any other race, is still a disease of poverty, and any significant increase in the economic status of the negro race would certainly result in a lower tuberculosis death-rate." Pitifully small income, poor housing and malnutrition, and all the concomitants of poverty combine, in Guild's opinion, against the negro.

It is the death-rate from tuberculosis which is so high in the American negro, and it is the death-rate, not the sickness incidence, which is held to depend on malnutrition. Recognizing the widespread incidence of the infection, attention should be directed to the mortality and invalidism which follow upon lowered resistance. Improvement in all the nutritional factors should be aimed at, and foremost amongst them must be placed adequate feeding.

It was recognized long ago that in underfed patients arrest of active tuberculosis is not to be expected. The problem of diet in tuberculosis may be considered from the standpoint of some interesting and important physiological observations.

### DIET IN TUBERCULOSIS.

The fever of tuberculosis does not behave like other fevers, and requires special consideration. Many cases of tuberculosis with high fever fail to show the greatly increased metabolic rate seen in other fevers. Yet in non-febrile tuberculosis there may, in severe cases, be an increase of from 20 to 36 per cent. Protein destruction is not so marked as in pneumonia or typhoid fever, and the specific dynamic action of protein is the same in tuberculosis as in normal health; whereas in other fevers where there is great protein destruction there is no increased metabolism, due to the usual specific dynamic action of protein. Thus, whilst in other fevers there is no ground for the old-fashioned idea that protein intake must be limited for fear of stimulating the fever, there are reasons for refraining from increasing the protein intake of tuberculous patients.

In tuberculosis both protein and carbohydrate will increase the volume of respiration as well as the total metabolism, an increase which is often undesirable in patients suffering from pulmonary tuberculosis.

For this reason the tuberculous patient does not need so large an increase of food as do other febrile patients (Shaffer and Coleman put the figure for other fevers at 60 to 80 calories per kilogram daily instead of the normal 35; this means from 3,500 to 5,000 total calories daily for a sick adult). Since fat does not increase the volume of respiration to the same extent as protein and carbohydrate, the large intake of fat insisted on by the older physicians has a scientific justification.

If the important factors, rest, fresh-air and contentment, are utilized to their full extent, much more may be expected of diet in tuberculosis. Increased weight is not always accompanied by improvement, but improvement in health will always lead to a gain in weight.

Contentment is one of the factors which weighs most heavily in nutrition. When we send our patients to sanatoria, when we keep them there for months and wonder why they do not make the progress that others make, it may be this factor of contentment that is lacking and is holding them back. All these nutritional factors are of importance. We do not know which will be the more important in a particular individual. In one it may be food, in another it may be rest, or it may be freedom from anxiety and worry, or in yet another it may be merely a question of a contented mind. These are some of the very complex factors in nutrition.

The food should be simple, easily digested, well-prepared, and appetizingly served. " Easily digested ! " This is a puzzling expression. We can read in books how long it takes a dog to do various things with the food it has eaten. But do these tables of times of digestion really apply to every individual human being ? Every physiologist and every doctor knows they do not. They may represent broad rules, but their application has to be found out afresh for every individual. The amount should be slightly more than the patient's calculated calorie requirement. For a man weighing 10 stone, who is up and about, some 3,000 calories may be necessary. The younger the patient the greater proportionately is the protein and calorie requirement. Here again caution is necessary. We take a patient's weight and surface-measurement and calculate that his or her requirement will be so many thousand calories. But the calculation is mightily inexact. There have been some very careful observations made on people with sufficient means to allow them to eat what they please: people whose size, age and work were approximately equal. The difference in their calorie requirements was simply amazing, and the observations were not made over short periods, they were long enough to show that each individual had a stable maintenance diet. We must remember, then, that all calorie requirements are average and that there is a wide range of variation in both directions.

The kind of protein matters; at least half should be first-class (animal) protein. Whilst the proportion of fat may be higher than in other diseases, not less than half the total calories should be derived from carbohydrate.

In order to ensure a sufficiency of vitamins and mineral salts the diet should include fruit, tomatoes, raw salads and green vegetables, as well as milk, butter and eggs. Milk and eggs are sometimes ordered to the exclusion of other foods, because it is easier to specify a given amount of each than to calculate the protein and calorie value of a more varied and more desirable diet.

But in health people do not really go on day after day drinking pints and pints of milk and eating nothing but eggs. It is an easy way of calculating things, but something better has to be thought out—there must be variety in the diet. Milk has an immense value, its calcium is needed for healing, but the patient must not be discouraged and even nauseated by its monotony.

A certain amount of roughage is essential, but the exact quantity must be determined by the comfort of the individual patient. Digestive disturbances are common in any case of tuberculosis, and ill-advised "stuffing" may precipitate such disturbances as well as induce great distaste for food of any kind. The best plan is to adopt a simple, well-balanced diet with no food between meals. A quart of milk daily is sufficient for an adult, and cooked eggs are better assimilated than raw. Resting before and after meals assists the appetite and the digestion.

I doubt whether cod-liver oil should be used as a routine in every case of tuberculosis. It may sometimes prove a valuable supplement, but there is always the possibility of impairing a patient's appetite for other essential foods.

In conclusion, I should say that whilst diet by itself does not arrest tuberculosis, without an adequate diet the disease is likely to run a most unfavourable course.

I have mentioned that the Clifton Hotwells had a great reputation for the cure of consumption. Let us take warning from the history of the Clifton Hotwells. The Clifton churchyards are full of memorials to young ladies who came here and died, not because they drank the Hotwells water, but because the disease of which they came to be cured was in such an advanced stage that not even the Hotwells water could arrest the disease. The result was that the Clifton death-rate rose so appallingly that people were scared. As a spa it failed, and for many years it was shunned as a place of residence. We must be careful how we advance any of our measures of cure as being infallible.

